# Concept confirmation of the Schizophrenia Cognition Rating Scale (SCoRS) among unpaid and professional caregivers

**DOI:** 10.1038/s41537-025-00674-2

**Published:** 2025-10-17

**Authors:** Christoph U. Correll, Sebastien Tulliez, Maggie Heinrich, Katja Rudell, Alessandra Girardi, Richard Keefe, Abraham Goldring, Corey Reuteman-Fowler

**Affiliations:** 1 Zucker Hillside Hospital, Department of Psychiatry, Northwell Health, Glen Oaks, NY USA; 2 Donald and Barbara Zucker School of Medicine at Hofstra/Northwell, Department of Psychiatry and Molecular Medicine, Hempstead, NY USA; 3 The Feinstein Institute for Medical Research, Center for Psychiatric Neuroscience, Northwell Health, New Hyde Park, NY USA; 4 Charité - Universitätsmedizin Berlin, Department of Child and Adolescent Psychiatry, Berlin, Germany; 5 German Center for Mental Health (DZPG), partner site Berlin, Berlin, Germany; 6 Boehringer Ingelheim International GmbH, Frankfurt, Germany; 7 Parexel International, London, UK; 8 Duke University Medical Centre, Durham, NC USA; 9 Episteme Inc. NY; Nathan S Kline Institute, Orangeburg, NY USA; 10 Boehringer Ingelheim Pharmaceuticals Inc., Ridgefield, CT USA

**Keywords:** Schizophrenia, Psychosis

## Abstract

Schizophrenia is a complex mental health condition characterized by a range of heterogenous symptoms. Cognitive impairment associated with schizophrenia (CIAS) interferes with individuals’ ability to manage their daily activities and has a detrimental effect on everyday life. The Schizophrenia Cognition Rating Scale (SCoRS) is an interview-based assessment, which was developed to provide a detailed evaluation of the performance of everyday tasks in the real world. The assessment is completed by clinicians with input from patients with schizophrenia and caregivers. The scale has good psychometric properties and has been used across several trials. Nevertheless, the relevance of the SCoRS from the caregivers’ perspective has not been confirmed. The aim of this study was to confirm the content of the SCoRS from the caregivers’ perspective. A cross-sectional qualitative study was conducted with primary (live-in, for example, a family member) and secondary (professionally trained) caregivers in the US caring for patients with schizophrenia. The caregiver research confirmed the concepts that clinicians and patients had previously identified in the creation of the PRECIS scale as clear cognitive deficits of patients with schizophrenia. Understanding that family and professional caregivers confirm these cognitive impairments to be real, independently from the patient and clinicians, is helpful to determine the validity of the cognitive impairment concept. Overall, the findings confirmed that the SCoRS captures relevant aspects of cognitive functioning in patients with schizophrenia and support the relevance and clarity of instructions, domains, and items with primary and secondary informants.

## Introduction

Schizophrenia is a severe and complex mental health condition starting early in life and is associated with significant morbidity, poor quality of life, and an enormously high burden of illness for 2.4 million adults in the US, with annual associated costs estimated to be more than 150 billion USD^1–4^.

Individuals with schizophrenia often experience persistent difficulties with their cognitive or thinking skills, such as learning, memory, problem-solving, verbal fluency, and speed of processing^5,6^. Cognitive impairment is one of the first signs of schizophrenia prior to diagnosis and extends throughout the time course of the illness^7^.

Cognitive impairment associated with schizophrenia (CIAS) is burdensome with detrimental effects on patients’ everyday life such as education, employment, independent living, and interpersonal relationships, thereby interfering with functional and life engagement goals^8–11^ Difficulties in daily functioning are associated with social isolation, exacerbated symptoms, mental health challenges, and potential relapses, as well as intensity and cost of care^7^. Additionally, people with schizophrenia have a reduced life expectancy^12,13^, and increased mortality risk due to unnatural and multiple natural reasons^7,14^.

The Schizophrenia Cognition Rating Scale (SCoRS) has been used as an endpoint in several international trials^15–17^. Compared to performance-based measures such as the MCCB and BACS, the SCoRS is an interview-based measure that assesses how various aspects of cognitive impairment affect the everyday life of the person with schizophrenia. The SCoRS includes the perspectives of the patient, caregiver, and clinician. Its psychometric properties, such as strong test-retest reliability, strong interrater reliability, treatment sensitivity, and internal consistency with a Cronbach’s coefficient alpha of 0.79, have been reported in several publications^15,16,18,19^. Although the assessment requires input from clinicians and caregivers (i.e., informants) regarding the impact of cognitive disabilities on the patients’ day-to-day functioning, the validity of the content from the informants’ perspective has to the best of our knowledge, not yet been investigated.

Therefore, the aim of this study was to assess the relevance and understanding of the SCoRS using qualitative interviews with primary (individuals who are taking informal, unpaid care of the patient, such as family members) and secondary (professionals with formal training in working with patients with schizophrenia) informants. Both caregiver types were included as they each bring specific strengths to the assessment. Professional caregivers can provide valuations with regards to other patients, and family caregivers can provide more thorough advice since they have seen decline/impairment development over longer time spans and often know the patient more intimately. The key objectives of the interviews were to conduct concept confirmation with informants to confirm that the SCoRS assessment captures the most relevant and meaningful aspects of cognitive functioning for individuals with schizophrenia and to conduct cognitive debriefing of the SCoRS to ensure understanding and clarity of the instrument for the informants. The outcomes of this study aim to determine the validity of the SCoRS concepts and the clarity and meaningfulness of the SCoRS items as provided by primary (PI) and secondary (SI) informants.

## Methods

### Study design

A qualitative cross-sectional non-interventional study, which was approved by the Salus Institutional Review Board (1346-0060), was conducted in the United States (US). Informants who cared for a patient with schizophrenia with a confirmed diagnosis participated in a one-on-one, 90-min, semi-structured interview. The sample of 20 patients is in line with minimum sample sizes for thematic and grounded theory analyses and was agreed as suitable for the study with the FDA^20^.

### Participants

Informants were classified into persons who lived with the participants or provided unpaid care and secondary providers of professional support and assistance, e.g., a social worker. Forty informants (20 primary caregivers; 20 secondary caregivers) were recruited between October 2021 and March 2023 via US patient/caregivers and professional association groups, or sites that participated in Phase 2 (1346.9) and Phase 3 (1346.38) clinical trials exploring treatment of cognitive impairment associated with schizophrenia. In terms of recruitment via clinical sites, only participants (informants) of patients not involved in the trials or who already completed the clinical trials were recruited to avoid potential confounding effects of treatment on the experience of disease.

No information on patient symptoms and degree of disability was collected in this study.

Informants who met the caregiver eligibility criteria (see Supplementary material S1) and provided informed consent were included in the study.

### Measures

The SCoRS is a 20-item interview-based clinical assessment used to evaluate the impact of cognitive impairments on patients with schizophrenia day-to-day functioning on eight cognitive domains: memory, learning, attention, working memory, problem solving, processing/motor speed, communication/social cognition, and language. The rater uses information derived from interviews with patients and informants (a person who has regular contact with the patient in everyday situations) and a rating generated by the interviewer who administered the scale. Items are rated on a 4-point scale with higher scores indicating a greater degree of impairment. Assessment takes approximately 15 min to be completed^16^. The scale total score (range: 20–80) was shown to be responsive to treatment effects^21,22^. The SCoRS has strong psychometric properties, including test-retest and inter-rater reliability, and sensitivity to treatment has been demonstrated in several studies^23^.

### Procedure

Trained researchers screened potential subjects for eligibility using a screening checklist and obtained informed consent via e-mail, telephone, and/or audio call. All interviews were conducted remotely via video call using MS Teams and were audio recorded. Two discussion guides were developed: one for the primary informants, and the other for the secondary informants. Each interview lasted approximately 90 min and included three parts:

**Part 1**: Informant and patient characteristics: informants’ socio-demographics and their relationship with the person diagnosed with schizophrenia. Informants were also asked to rate the severity of cognitive impairment of the person with schizophrenia they were caring for in the previous two weeks on a 4-point rating scale (none, mild, moderate, severe).

**Part 2**: Concept confirmation: to evaluate if the content of the SCoRS adequately covered the most relevant and meaningful aspects of cognitive functioning in the schizophrenia patient population. Informants were interviewed about their experience of caring for or spending time with patients with schizophrenia.

Part 3: Cognitive debriefing: to evaluate understanding and clarity of the SCoRS instructions, items, response options, and the recall period.

### Data analysis

In this study, 20 participants were recruited for each informant group, to facilitate a qualitative comparison of primary and secondary informants’ experience.

Interview transcripts were de-identified and uploaded into Atlas.ti, a qualitative software package which facilitates the organization and analysis of qualitative data^24^.

A coding dictionary was developed based on the discussion guide. The codebook was modified, when required, based on the concepts raised by participants during the interviews.

Two researchers held regular meetings where they discussed, reviewed, and updated the codes. The iterative refinement of codes continued for each interview individually until all 40 interviews were reviewed. The coding process was conducted in accordance with industry guidance on qualitative research methods^25^. To ensure quality in the data coding process, approximately 20% of the transcripts were parallel coded by two researchers. The point when “saturation” (defined as the point when no new information is discovered in data analysis^26^) has been achieved was monitored throughout the study by populating a tabular summary of concepts endorsed by interview participants. The a priori threshold to identify relevant concepts was set at ≥30% of endorsement^27^. The minimum threshold for endorsement was set a priori by the outcomes researchers in the Qualitative Analyses Plan (QAP). Considering the cognitive heterogeneity in schizophrenia and thus varied manifestations of the cognitive impairment in patients, an “endorsed concept” was defined as one that was endorsed by a minimum of 30% (*N* = 12) of the caregiver sample.

The cognitive debriefing part of the interview was also analysed following standard procedures^25^, to evaluate participants’ understanding of the SCoRS.

Means, ranges, and frequencies were computed to summarize the informant and patient characteristics.

## Results

### Sample characteristics

Most informants were female in both primary and secondary informant groups (85% and 60%, respectively) and held a higher education degree (Bachelor's or Master's). Primary informants were older (mean age = 51.2; range = 28–82) compared to secondary informants (mean age = 40.9; range = 22–60) (Table 1).

**Table 1:** Informant characteristics and relationship with patients.

Informant characteristics	Primary informants *N* (%)	Secondary informants *N* (%)
Age (years)		
19–25	0 (0)	2 (10)
26–34	5 (25)	3 (15)
35–54	5 (25)	13 (65)
55–64	4 (20)	2 (10)
65+	6 (30)	0 (0)
Gender		
Male	3 (15)	8 (40)
Female	17 (85)	12(60)
Education		
Bachelors	9 (45)	6 (30)
Masters	8 (40)	9 (45)
College	2 (10)	2 (10)
High school	1 (5)	N/A
PhD	N/A	3 (15)
Relationship to the patient*		
Parents/other relatives	18 (90)	N/A
Partners	2 (10)	N/A
Social worker	N/A	8^a^ (40)
Therapist	N/A	5^a^ (25)
Clinical research coordinator	N/A	3^b^ (15)
Other		7 (35)
Length of time interacting with the patient (years)	*N* = 19	*N* = 20
0–5	5 (25)	18 (90)
6–10	4 (20)	1 (5)
11–15	1 (5)	1 (5)
16–20	5 (25)	0 (0)
21+	4 (20)	0 (0)

*N/A* not applicable.

^a^One was a social worker and therapist.

^b^One secondary informants was sub-investigator and rater; total *N* = 19 as one participant mentioned they have known their patient “Since birth” without indication of a specific length of time.

Most primary informants were relatives of patients with schizophrenia (90%). Individuals in the secondary information group had multiple roles, with the majority (40%) being social workers. On average, primary informants reported longer interaction time with the patients they were currently caring for (mean = 14.6 years; range = 1–35 years) compared to professional informants (mean = 7.0 years; range ≤ 1 year – 13), with an overall experience of working with individuals with schizophrenia ranging from less than 1–30 years (mean = 11.4).

Primary informants described patients’ cognitive impairment from none to severe, with the majority having mild, moderate, and severe impairment, whereas patients cared for by the secondary informants were described as mild or moderately impaired.

Most of the patients (90%) who were cared for were taking antipsychotic medications at the time of the informants’ interview and less than 50% were hospitalized for schizophrenia in the previous year (see Supplementary Material S2).

### Concept elicitation

Saturation was achieved, based on most concepts being reported by ≥30% (of the primary and secondary informants.

#### Item level endorsement

Concepts reported spontaneously (S) or prompted (P) during the concept elicitation part of the interview are shown in Table 2:

**Table 2:** Concept confirmation table for primary (PI) and secondary informants (SI).

	Primary informants			Secondary informants		
Concepts	“S” %	“P” %	TOTAL %	“S” %	“P” %	TOTAL %
**Memory deficit**						
1. Names of people	5%	35%	40%	5%	40%	45%
2. Direction to places	0%	45%	45%	25%	25%	50%
4. Location of objects	15%	50%	65%	20%	60%	80%
5. Task/Chores	45%	25%	70%	50%	15%	65%
Orientation to time (new theme)	0%	0%	0%	10%	0%	10%
**Learning deficit**						
6. Using new gadgets and equipment	0%	65%	65%	15%	50%	65%
14. Learning new things / new ways of doing things	5%	70%	75%	0%	40%	40%
**Attention deficit**						
3. Following a TV show	30%	40%	70%	0%	45%	45%
11. Concentrating well enough to read	20%	35%	55%	0%	35%	35%
13. Staying focused	50%	40%	90%	30%	30%	60%
20. Attention in conversation	10%	0%	10%	15%	0%	15%
**Working memory deficit**						
7. Remembering recent information	35%	20%	55%	35%	55%	90%
8. Train of thought	10%	45%	55%	5%	45%	50%
**EF/problem solving deficit**						
9. Keeping track of money	5%	60%	65%	30%	20%	50%
12. Completing a familiar task	30%	55%	85%	30%	25%	55%
17. Handling changes in routine	0%	50%	50%	10%	40%	50%
Other/general problem-solving deficit	15%	0%	15%	10%	0%	10%
**Processing deficit**						
15. Speaking speed	0%	35%	35%	10%	55%	65%
16. Task speed	0%	35%	35%	0%	35%	35%
Other/general processing deficit	15%	0%	15%	5%	0%	5%
**Communication/social deficit**						
18. Conversational confusion	25%	60%	85%	35%	35%	70%
19. Reading emotion	0%	55%	55%	0%	50%	50%
20. Following conversations in a group	10%	45%	55%	15%	45%	60%
Other/general communication and social deficit	5%	0%	5%	5%	0%	5%
**Language deficit**						
10. Jumbles words	5%	35%	40%	5%	25%	30%
18. Language difficulties leading to conversational confusion	10%	0%	10%	15%	0%	15%
20. Participating in a group conversation	0%	40%	40%	0%	40%	40%
Other/general language deficit	5%	0%	5%	5%	0%	5%

*S* spontaneous, *P* prompted.

Across the items of the SCoRS, there was high endorsement ≥30%; e.g., Nelsen et al.^27^ of most concepts (18/20), among both PIs and SIs alike. Both informants observed a notably high extent (i.e., ≥50%) of difficulty in: remembering the location of objects (Item 4; PI 65%, SI 80%), tasks/chores (Item 5; PI 70%, SI 65%), using new gadgets and equipment (Item 6; PI 65%, SI 65%) staying focused (Item 13; PI 90%, SI 60%) remembering recent information (Item 7; PI 55%, SI 90%), train of thought (Item 8; PI 55%, SI 50%), keeping track of money (Item 9; PI 65%, SI 50%), completing a familiar task (Item 12; PI 85%, SI 55%), handling changes in routine (Item 17; PI 50%, SI 50%), conversational confusion (Item 18; PI 85%, SI 70%), reading emotion (Item 19; PI 55%, SI 50%), and following conversations in a group (Item 20; PI 55%, SI 60%).

The two items that were not endorsed by more than 30% of the sample sub-groups (PIs, SIs) included: language difficulties leading to conversational confusion (Item 18; PI 10%, SI 15%) and attention in conversation (Item 20; PI 10%, SI 15%).

**Fig. 1:**
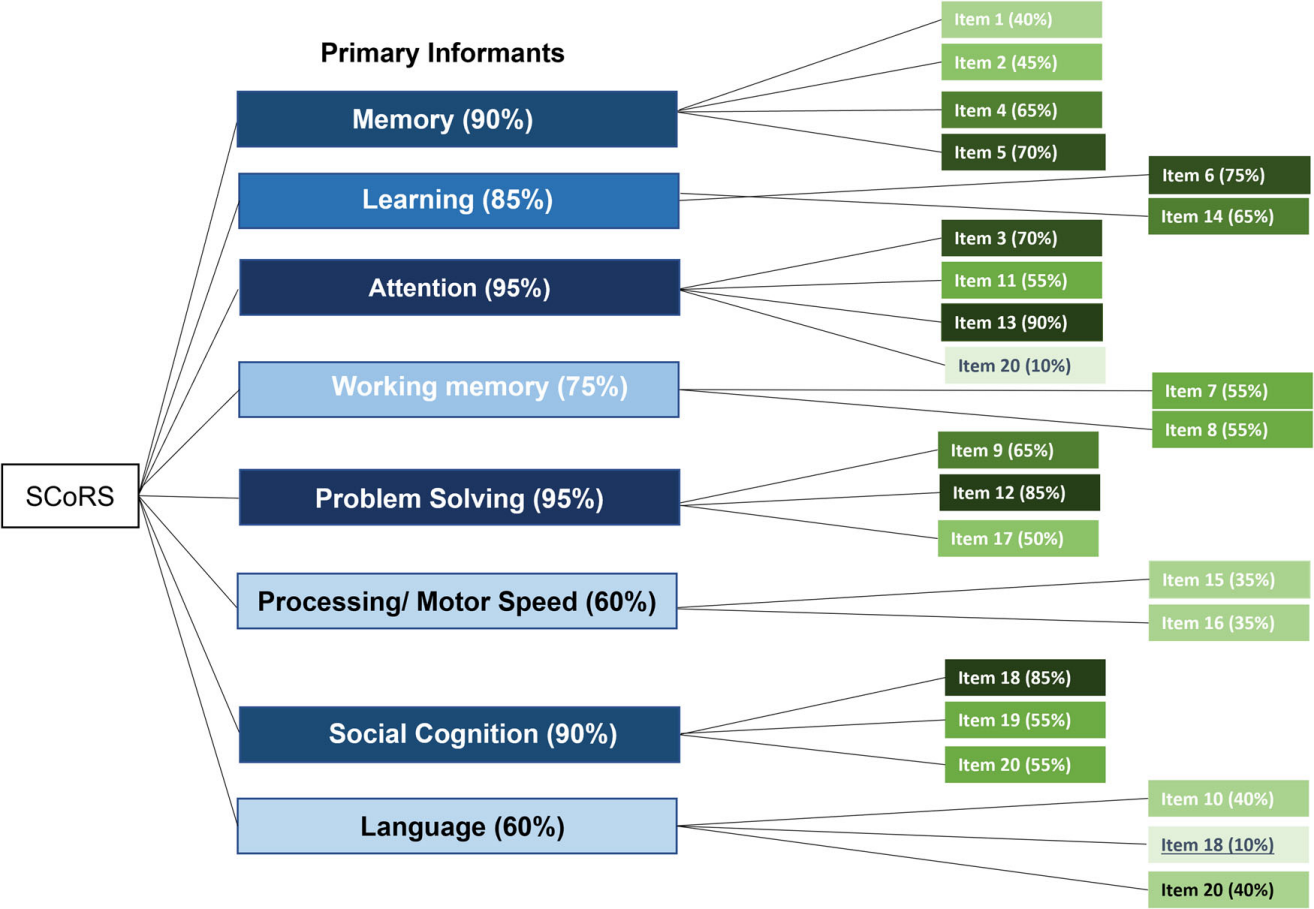
Mapping of items by % endorsed by PIs onto the SCoRS. The highest endorsed items are marked with a darker color and the lowest endorsed domains are marked with lighter shading.

**Fig. 2:**
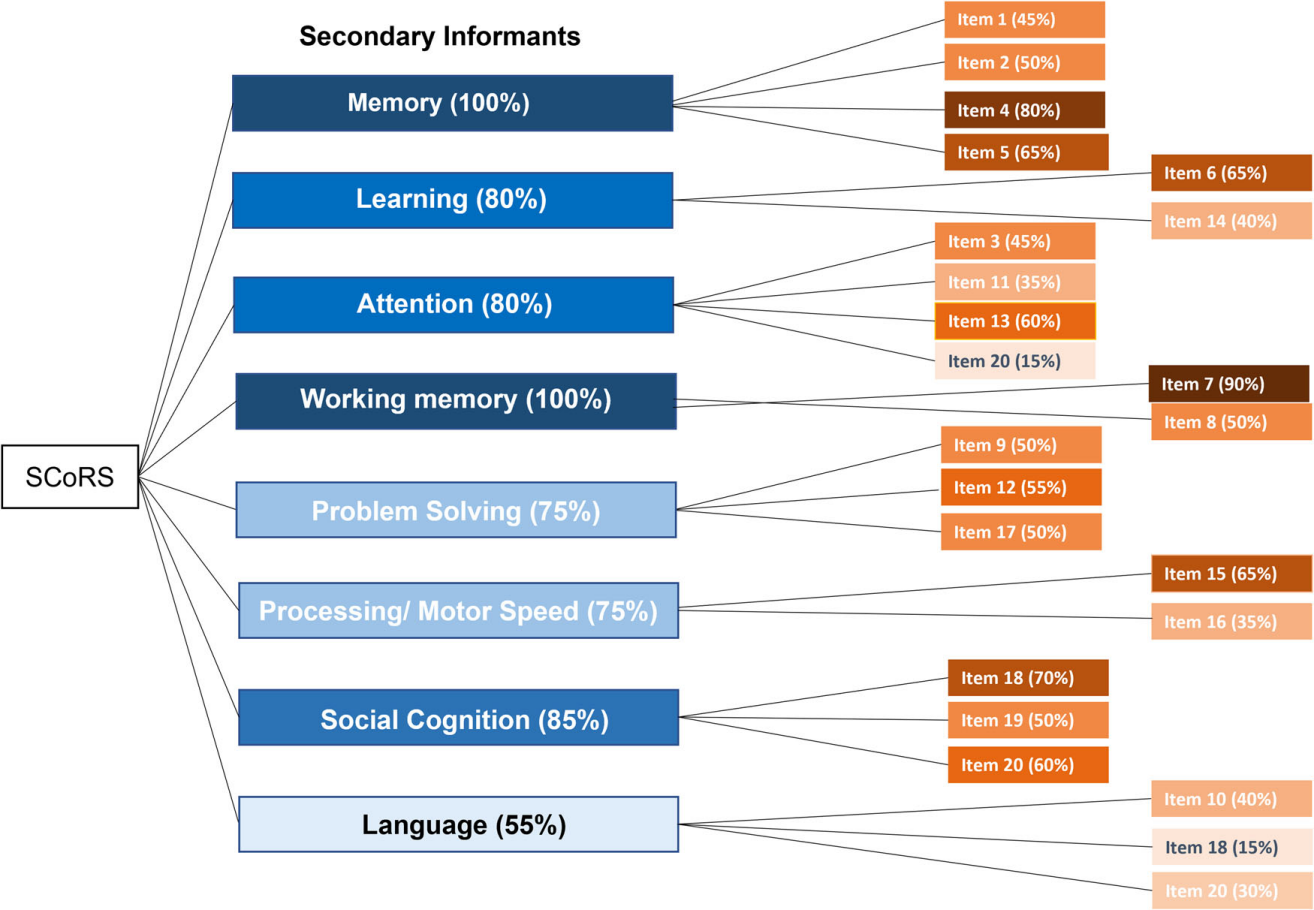
Mapping of items by % endorsed by SIs onto the SCoRS. The highest endorsed items are marked with a darker color and the lowest endorsed domains are marked with lighter shading.

The concepts elicited by PIs and SIs (at an item-level) were mapped onto the SCoRS instrument (Fig. 1 for PIs and Fig. 2 for SIs). Staying focused (90%), within the attention domain was the highest endorsed domain for PIs and remembering recent information (90%), within the working memory domain was the highest endorsed domain for SIs. Similarly, in both informant groups, understanding what people mean when they are talking to them, within the language domain was the least endorsed domain for both groups (with 10% of PIs reporting this and 15% of SIs reporting this).

#### Item analysis

All of the content of the SCoRS was endorsed by informants, with most (18/20; 90%) confirmed by both primary and secondary informants (see representative quotes in Table 3 below).

**Table 3:** Exemplary quotations of cognitive symptoms elicited in a spontaneous and probed manner.

Concept	Domain	Exemplary quotations
Names of people (Item 1)	Memory	P14 (Spontaneous): “Basically, he has a lot of problems, let’s say, with memory. **He comes to a point when he can’t even remember the names of the family members sometimes**, and there are a lot of hallucinations.” S3 (Probed): “He and I have been working together for the past two years and **he often times forgets my name, particularly my last name**. Phonetically, he can say my name, but he wouldn’t be able to spell it. If this makes any sense, he understands the rhythm of my name.”
Direction to places (Item 2)	Memory	P11 (Probed): “She’s very good with people. Places, yes. Sometimes, when the situation… And you have to, somehow, we manage. But a lot of times, I can’t tell because I’m with her and I do some of that. And **she’s not as quick as I am, I don’t think, to find her way in terms of getting to this place or that place**.” S3 (Spontaneous): “My patient has a **difficult remembering locations**, dates and times.”
Location of objects (Item 4)	Memory	P1 (Spontaneous): “(…) He **constantly loses things** because he **doesn’t remember where he put it**. He’s constantly losing things to the point where he blames me for having moved somethings that I never touched because he doesn’t even remember that he put it where it is.” S6 (Spontaneous): “Or the one time they did call they **did not get through and then lost the post-it again**. And this has happened will happen.”
Task and Chores (Item 5)	Memory	P8 (Spontaneous): “So he also **struggles to remember to keep his appointments with his healthcare providers**. He does seem to not forget when I’m coming, because I take him shopping every week, and that he doesn’t forget about. But when he has other appointments, he often forgets about those.” S12 (Spontaneous): “Oh, yeah. Well, I mean, just directly, we would have an appointment scheduled for, say, Thursday at 9:00 a.m. and 9:05, 9:10, they’re not here. 9:30, okay, we call. **Even though they had already received a call the day before, that they had the appointment at 9:00, but that they had forgotten about the appointment**. So, usually, the subject’s been okay coming in afterwards, but they had to get a second call from us to remind them that they had already missed their appointment, and this has happened for numerous appointments.”
Using new gadgets and equipment (Item 6)	Learning	P12 (Probed): “Yes. Yes, let’s say for example, I change the mobile phone I’m using, most probably she will not even know where to start, where to unlock. And sometimes, I tend to **think it’s because maybe she perceives it’s a new item she’s not used to, not because it is complicated** or what, but just a slight difference from the previous one will confuse her.” S10 (Spontaneous): “There is an app that’s related to the investigatal^1^ product that we give to the patients, and there’s a device that we hand out in the beginning of the trial. And the **subject has many issues using the device, working a device, and actually using it on a daily basis as he is required to**. So, there’s been many instances where if **I had to reeducate the subject on how to use the device**, and there 9, 10 months that he was in the trial, it progressively got worse. And then at one point, he was pretty non-compliant with not using the actual device.”
Learning new things/new ways of doing things (Item 14)	Learning	P12 (Spontaneous): “You said learning is part of cognitive. And then you will find also, if it’s a game you are teaching her, you’ll have to repeat so many times such that… My kid who is 6 years old can even capture the concept more than she does. **So, it will take a long time for her to learn how it is being played and to play on her own. So, I can say she learns very slowly**.” S19 (Probed): “Learning to do activities here, it may be something new for him, or transition, schedule changes. **In order for him to be comfortable, or to get him to follow through and be cooperative**, it really would need to be someone that directly works with him, that’s helping him to learn this or to make that change. Or **if not, he’ll refuse and he won’t do it**.”
Following a TV show (Item 3)	Attention	P3 (Spontaneous): “Some of the problems I observed, when he’s bored, he’ll say there’s nothing to do. I’ll say remember you used to like such and such show. **But he’s not able to watch TV. Like he can’t concentrate long enough or settle his mind enough to actually pay attention to the show or program**” P12 (Spontaneous): “And also, even paying attention. Sometimes, we are all together and we are watching maybe a game, or a movie. And you find **when others are maybe celebrating, maybe the team you’ve been supporting has won. As for her, she just forgot we were watching or maybe she cannot concentrate for a long time**.”
Concentrating well enough to read (Item 11)	Attention	P3 (Spontaneous): “I find the same thing when I suggest books or comics or the newspaper, **it’s like his concentration, his thoughts are always kind of racing so it’s hard for him to bring himself back and settle down and watch that TV show or read that book**.” S17 (Probed): “**I would say probably some deficits with being able to keep focused on what they’re reading to the point of comprehension**, yeah. I don’t see the person read very much.”
Staying focused (Item 13)	Attention	P11 (Spontaneous): “Concentration is difficult, especially sometimes in certain cases, whether it’s conversation, or just sort of focusing in on one **particular type of topic, and really focusing on one thing**. **So sometimes, it’s a little scattered, it feels**.” S18 (Spontaneous): “**He has difficulty with focusing. We have to always assist him, and use prompts**, and helping him to be able to answer questions, and to be able to process what is being said to him.”
Attention in conversation (Item 20)	Attention	P8 (Spontaneous): “Well, I guess **he has a hard time following what other people are saying in conversations. I think his thoughts are just racing so much**, and he just talks incessantly, and doesn’t generally listen much. He has a hard time when focusing, and even **I think he has a hard time staying on track with his own thoughts because he’s bouncing all over the place**.” S17 (Spontaneous): “Probably related to attention stuff. If they’re not being spoken to directly, **staying engaged in a conversation when they’re not being prompted directly or spoken to directly, they tend to drift off**.”
Remembering recent information (Item 7)	Working memory	P13 (Spontaneous): “She also has a problem – she can learn something right now, like normally, you expect someone to learn something. **Once they have understood, they can implement it. She has a problem with using information immediately after she has learned it**.” S16 (Spontaneous): “And she has to be repeated things. **If you explain things to her and say, “Tell me what you heard me say,” she doesn’t give it back right**. ”
Remembering what they were going to say (Item 8)	Working memory	P16 (Spontaneous): “Another thing that she’ll make note of is if she is going to – well, this is going to be more of a past thing, because she doesn’t use her phone to call anybody anymore – but **when she was going to be calling somebody, she would write out what she was going to say to start out**, you know, whether she was making a complaint or asking for something, whoever she needed to talk to, she would have like a little script to get her through it.” S8 (Probed): “Yeah, one that comes to mind is forgetting what **they were going to say or not remembering, and that can be frustrating for the person**. I have to just sit back and not to jump in to try to help the person. I notice that is frustrating, yeah. So forgetting I guess, what was it, names, and thoughts, **losing their sequence of thoughts seems it could be problematic**.”
Keeping track of money (Item 9)	Executive function	P6 (Probed): “Yes, he can keep track of money, he can count change. **He’s not good with money, he spends money a lot. He spends it very foolishly**, but I think he can definitely count it and make change” S2 (Spontaneous): “So, like most of us know to budget our money. We know you have to pay this money for this bill and that bill. **This individual would just spend all their money up**. Not understand that you might need money for tomorrow or not all the money is for you. You have to have money for your rent, you need money for food, you need money to buy clothes and other things. **They don’t understand that they need to save some money so they will spend all their money up in one shot**.”
Completing a familiar task (Item 12)	Executive function	P9 (Spontaneous): “I don’t know if this is important or not. **He has a hard time cooking for himself**. I just think it’s hard for him with all the medications he’s on and working nights, but I don’t know if that is anything that would be helpful. But I know to teach him to cook, it’s kind of hard to do.” S19 (Spontaneous): “First thing that stands out is **just self-care, remembering to take baths, brush his teeth, change clothes**. He can start a task with assistance, but it’s hard finishing tasks. But we do transport our clients, and so it’s better now.”
Handling changes to daily routine (Item 17)	Executive function	P18 (Probed): “Having to maybe change a schedule where he has to go somewhere at a different time. (…)**. Any type of change involving having to let’s say even get dressed in the morning. If you change the time that he normally would get up to a different time, and just getting on that schedule might be a little bit difficult for him to do**.” S2 (Spontaneous): “Sometimes **they don’t have ability to balance out their daily routine**. Like I said, they don’t keep up their appointments because they forgot they had their appointment or they didn’t realize that they needed to come on a certain day.”
Speaking speed (Item 15)	Processing/motor speed	P18 (Probed): “I can routinely ask him about something that I may have asked him to do. For instance, I can say, did you feed the dog? And then he’ll go**, “mmm…yes.” It’s that much delayed, or maybe even longer sometimes, because I may have to repeat myself**.” S15 (Probed): “**He does have very slow speech and a lot of pauses**. I will confirm that. (…) Just usually, whenever engaging with him, **it takes him a long time to get what he needs to say out**. Or once again, like I said, he gets stuck on certain words. So it takes him a long time to process that. **He generally isn’t a fast-moving person, so sometimes he’s just very slowed or delayed in responses**”
Task speed (Item 16)	Processing/motor speed	P13 (Probed): “**Moving slowly, she does**. Yes, she does.” S15 (Probed): “**He is a slow writer**. But as far as his other tasks, I would say he’s about normal speed.”
Understanding how other people feel about things (Item 19)	Communication/social cognition	S7 (Probed): “(…) **I don’t think he does have a good connection to how the other people around him feels when he’s expressing himself in certain areas**, because he just – he will be just communicating, and talking, and saying things without allowing the other person to kind of engage with him, in a way, unless you interrupt and say, “Hey, you know,” kind of interrupt him a little bit.” S16 (Probed): “She’s not a good…**she doesn’t read well with people**. She does not pick up on a person’s…if a person is angry, she may think that they’re a little bothered, but **she doesn’t pick up when a person is just so angry** that they could hurt her. I don’t know if she just minimizes it.”
Following conversations in a group (Item 20)	Communication/social cognition	P16 (Spontaneous): “Currently, she pretty much spends all her time alone. It’s what she prefers. We do find when we try to interact with her, **she has a canned response to us, so what she has developed is always pretty much the same, therefore she doesn’t really have to listen to what we say and process it**, she just blurts out, so even if you ask a question, you’re still going to hear the same canned response. We try to slow her down and say, “No, this is what we are asking you, we need an answer for this.” I guess maybe you need to ask me again what you want.” S12 (Spontaneous): “(…) just if there’s more – **even with just one person talking to him, you often have to repeat yourself, rephrase questions, break them down into easier to remember terms**. And if he’s in a room with more than one person, if all three or four people are talking, he often gives you like a blank stare, or cannot kind of grasp what the conversation’s about. If a question is asked of him, he’ll just kind of look at you and he’ll need to be walked through the question.”
Keeping their words from being jumbled together (Item 10)	Language	P7 (Spontaneous): “The other thing is he **can’t verbalize**. **He thinks normally to a large extent, but he can’t verbalize it. When it comes out, it comes out all garbled, and then people look at him very strangely**. (…) But his verbal – I mean, he uses big words. His vocabulary is very good. But the processing, I don’t know. I don’t know what happened.” S9 (Spontaneous): “You know, I don’t know if they’re just speaking what’s coming off the top of their tongue, just a ton of racing thoughts, a ton of just – you know, **different thoughts jammed together into one**”
Participating in a group conversation (Item 20)	Language	P6 (Probed): “With the group conversations. He doesn’t have word salad. He doesn’t mix up his words and he doesn’t have any of that, thank God. **But with the group conversations, yeah, like when his friend comes over, his other friend came over that he’s known for a little while**. And the three of us were trying to have a conversation. And again, I wound up having more of a conversation with his friend than he did. **It was kind of hard for him to have a conversation with the three of us**. And like I said months ago, when his other friend had come over, **it was so hard for him to converse with his friend**, even one on one while I was there. So yeah, with that, definitely, having difficulties with conversation. **Because in everyday life, you have to converse with people, and he has a hard time there sometimes**.” S16 (Probed): “**She again will oftentimes not talk, if it’s something that she doesn’t know about. She just doesn’t do a lot of talking in group settings**. If you call on her name…say for instance if it’s a group therapy and you call her name to ask her feedback on a topic, she may give something very vague or something that’s unrelated to the topic.”
Understanding what people mean when they are talking to them (Item 18)	Language	P20 (Spontaneous): “She really don’t say much. You ask her a question, or give her a command, it’s like okay, and **she really don’t say much**.” S18 (Spontaneous): “So, the thinking, being able to process what is being said to him, he gets very agitated when people call him about bills because he’s **unable to answer them or be able to speak what he’s trying to say while they can understand him**.”

Main concepts from the quotations are marked in bold.

*P + Number* primary informant identification number, *S + Number* secondary informant identification number.

### Cognitive debriefing

#### General instructions debriefing (including recall period)

There was a high level of understanding among both primary (100%) and secondary (85%) informants, which was reflected with both informants reporting 100% ease of understanding. When informants were asked about the ease of thinking about the past two weeks, the majority of the primary (75%) and secondary (90%) informants confirmed it was easy.

Five PIs reported difficulty with the recall period. For example, P12 said *“I can say somewhat difficult because it means I have to trace, maybe sometimes, using activities, what we have been doing the last 2 weeks. But maybe sometimes, you find you have been having so many affairs to run, and sometimes you can’t remember*.” Full quotes are shown in Supplementary Material S3.

#### Cognitive debriefing of SCoRS items

Across the items of the SCoRS, there was high (85%–100%) correct interpretation, ease of response option selection and the examples considered helpful for the majority of the items. Overall, a lower proportion of PIs correctly interpreted the items and mentioned that it was easy to select response options for the items compared to SIs.

There was a lower proportion of PIs that correctly interpreted Item 16 than the SIs. Six primary informants (30%) incorrectly interpreted Item 16, trouble completing tasks quickly.

For the ease of response selection, five primary informants (25%) stated it was difficult to select a response option for item 1, remembering names of people. Six primary informants (30%) reported that it was difficult to select a response selection for Item 16. Finally, five primary informants (25%) stated it was difficult to select a response selection for Item 19, difficulty understanding how other people feel.

When asked if examples are helpful for each item, ≥80% of the primary informants mentioned that the examples were helpful for all items except for item 16. Five primary informants (25%) noted the examples were unhelpful for this item. The examples for all of the items were considered helpful by a high percentage (85%–100%) of the secondary informants.

#### Cognitive debriefing analysis of SCoRS items

The understanding of items, the ease of response selection, and the helpfulness of examples were comparable between primary and secondary informants. A high proportion of PIs and SIs (70%–100%) accurately interpreted the items, describing the response options as easy to use, and the examples as helpful.

Nevertheless, a trend emerged for a slightly lower proportion of PIs to accurately engage with items across all domains. The “ease of response option” appeared to be particularly impacted. The reporting of the following sections mirrors item appearance in the conceptual framework (Table 4).

**Table 4:** Cognitive debriefing of items in the SCoRS by primary and secondary informants.

Aspect of debriefing	Primary informants	Secondary informants
	Agreement *n*/*N*^a^ (%)	Agreement *n*/*N*^a^ (%)
Item 1 “Remembering names”		
Correct interpretation	17/20 (85.0%)	19/20 (95.0%)
Ease of response selection	15/20 (75.0%)	19/20 (95.0%)
Examples considered helpful	15/18 (83.3%)	19/19 (100.0%)
Item 2 “Remembering how to get places”		
Correct interpretation	18/20 (90.0%)	20/20 (100.0%)
Ease of response selection	16/20 (80.0%)	20/20 (100.0%)
Examples considered helpful	18/19 (94.7%)	19/20 (95.0%)
Item 3 “Following a TV show”		
Correct interpretation	20/20 (100.0%)	18/20 (90.0%)
Ease of response selection	16/19 (84.2%)	19/20 (95.0%)
Examples considered helpful	20/20 (100.0%)	18/19 (94.7%)
Item 4 “Remembering where they put things”		
Correct interpretation	20/20 (100.0%)	20/20 (100.0%)
Ease of response selection	17/20 (85.0%)	16/20 (85.0%)
Examples considered helpful	20/20 (100.0%)	19/19 (100.0%)
Item 5 “Remembering their chores and responsibilities”		
Correct interpretation	18/20 (90.0%)	20/20 (100.0%)
Ease of response selection	18/19 (94.7.%)	19/20 (95.0%)
Examples considered helpful	20/20 (100.0%)	17/19 (89.5%)
Item 6 “Learning how to use new gadgets and equipment”		
Correct interpretation	20/20 (100.0%)	19/20 (95.0%)
Ease of response selection	17/20 (85.0%)	19/20 (95.0%)
Examples considered helpful	19/20 (95.0%)	19/20 (100.0%)
Item 7 “remembering information/instructions recently given to them”		
Correct interpretation	18/20 (90.0%)	18/20 (90.0%)
Ease of response selection	18/20 (90.0%)	20/20 (100.0%)
Examples considered helpful	20/20 (100.0%)	17/17 (100.0%)
Item 8 “Remembering what they were going to say”		
Correct interpretation	17/20 (85.0%)	19/20 (95.0%)
Ease of response selection	19/20 (95.0%)	19/20 (95.0%)
Examples considered helpful	19/19 (100.0%)	19/19 (100.0%)
Item 9 “Keeping track of their money”		
Correct interpretation	19/20 (95.0%)	19/20 (95.0%)
Ease of response selection	17/20 (85.0%)	19/20 (95.0%)
Examples considered helpful	19/19 (100.0%)	15/16 (93.8%)
Item 10 “Keeping their words from being jumbled together”		
Correct interpretation	18/20 (90.0%)	19/20 (95.0%)
Ease of response selection	20/20 (100.0%)	19/20 (95.0%)
Examples considered helpful	17/18 (94.4%)	17/17 (100.0%)
Item 11 “Concentrating well enough to read a newspaper/book”		
Correct interpretation	17/20 (85.0%)	17/20 (85.0%)
Ease of response selection	16/19 (84.2%)	19/20 (95.0%)
Examples considered helpful	20/20 (100.0%)	17/17 (100.0%)
Item 12 “Familiar tasks”		
Correct interpretation	18/20 (90.0%)	19/20 (95.0%)
Ease of response selection	17/20 (85.0%)	20/20 (100.0%)
Examples considered helpful	18/19 (94.7%)	18/18 (94.4%)
Item 13 “Staying focused”		
Correct interpretation	16/20 (80.0%)	20/20 (100.0%)
Ease of response selection	17/20 (85.0%)	20/20 (100.0%)
Examples considered helpful	20/20 (100.0%)	18/18 (100.0%)
Item 14 “Learning new things”		
Correct interpretation	18/20 (90.0%)	19/20 (95.0%)
Ease of response selection	16/20 (80.0%)	20/20 (100.0%)
Examples considered helpful	18/18 (100.0%)	18/18 (100.0%)
Item 15 “Speaking as fast as they would like”		
Correct interpretation	20/20 (100.0%)	17/20 (85.0%)
Ease of response selection	18/20 (90.0%)	19/20 (95.0%)
Examples considered helpful	18/19 (94.7%)	18/18 (100.0%)
Item 16 “Doing things quickly”		
Correct interpretation	14/20 (70.0%)	16/19 (84.2%)
Ease of response selection	14/20 (70.0%)	20./20 (100.0%)
Examples considered helpful	15/20 (75.0%)	19/19 (100.0%)
Item 17 “Handling changes in their daily routine”		
Correct interpretation	17/20 (85.0%)	19/20 (95.0%)
Ease of response selection	17/20 (85.0%)	20/20 (100.0%)
Examples considered helpful	17/18 (94.4%)	18/18 (100.0%)
Item 18 “Understanding what people mean when they are talking to them”		
Correct interpretation	19/20 (95.0%)	19/20 (95.0%)
Ease of response selection	18/20 (90.0%)	19/20 (95.0%)
Examples considered helpful	17/19 (89.5%)	17/18 (94.4%)
Item 19 “Understanding how other people feel about things”		
Correct interpretation	19/20 (95.0%)	19/20 (95.0%)
Ease of response selection	16/20 (80.0%)	19/20 (95.0%)
Examples considered helpful	20/20 (100.0%)	18/18 (100.0%)
Item 20 “Following conversations in a group”		
Correct interpretation	19/20 (95.0%)	20/20 (100.0%)
Ease of response selection	16/20 (80.0%)	20/20 (100.0%)
Examples considered helpful	19/19 (100.0%)	18/18 (100.0%)

^a^In cases where the participant was not asked or did not answer the question, this is reflected in the denominator.

## Discussion

The results presented in this report confirm the content and the usability of the SCoRS from the view of caregivers. The caregiver perspective adds a further independent perspective that can enhance the evaluation of a patient with cognitive impairment and limited insight into their condition. Family members’ perspective as a caregiver may be confounded by past experiences, expectations, and relational aspects. In contrast, professional caregivers may offer a more unbiased opinion, yet generally more limited knowledge of and shorter times spent with the patient may create other biases. Hence, both types of informants can provide relevant information for the rating of the SCoRS. With regards to concept confirmation, all SCoRS concepts were endorsed by a high proportion (≥30%) of both primary and secondary informants. Items were endorsed either spontaneously or following probing by the majority of informants in both groups. Most items (*n* = 18; 90%) met standard criteria for endorsement (≥30%; e.g.,^27^). Two items (Item 18 and Item 20) were endorsed less frequently by both informant groups (endorsement ≤15%), both of which are from the communication/social cognition domain. These 2 items could not be observed by informants during the COVID pandemic and depend also on the nature of the caregiving, as not all of the patients’ impairments will be observable by professional caregivers. The challenges that people with schizophrenia face with communication/social cognition mean that they are less likely to engage socially^28^, and it is therefore not surprising that these two items were the least endorsed. Furthermore, those items that were 30%–50% endorsed included behaviors that manifest during social interactions (e.g., remembering names, attention in conversation, speaking speeds, and participating in conversation). These findings support the view that patients with schizophrenia often display poorer social skills and report fewer close relationships compared to patients without schizophrenia^18,29^. People with schizophrenia patients avoid contact with others, may feel easily criticized, and are sensitive to sounds, or variable speaking noises. In line with this view, the evidence indicates that annual rates of loneliness among individuals with schizophrenia and other psychotic disorders are 2.3 times higher (76–80%) compared to the general population (35%)^30,31^.

With regards to the cognitive debrief, overall, most informants (70%–100%) demonstrated an accurate understanding and use of the SCoRS. A trend emerged for a slightly lower proportion of PIs to accurately engage with items across all domains, particularly regarding the ease of response selection. However, some difficulties appeared related to the clinical profile of some individuals with schizophrenia (e.g., rare or no social relationships, no change of routine) or the uncertainty concerning what the patient is really thinking/understanding (e.g., no overt manifestations), rather than the scale properties. Secondary informants were trained professionals and therefore had limited exposure to the individuals, whilst seeing a larger variety of patients. The other patients they cared for and their exposure may affect the responses they provided, whereas, familial caregivers were probably closer focused on the individual case examples.

Finally, analysis of the quotes suggests that the cognitive impairment observed in patients with schizophrenia has a severe negative impact on caregivers’ activities (e.g., providing continuous support) and patients’ quality of life, including mood (e.g., feeling of frustration, agitations), social relationships (e.g., isolations), and treatment adherence (e.g., forgetting medicines, appointments).

It could be argued that the informants’ reported experience with this patient population would vary according to the observed level of disease severity. Objective assessment of disease severity would allow to account for individual difference. However, due to the interview-based approach used in this study, patients’ characteristics (e.g., severity of cognitive impairment) were only provided at a qualitative level by their primary and secondary informants. Nevertheless, all relevant concepts were discussed with all informants, either spontaneously or following prompting, and the findings indicate that items reached the standard threshold for saturation, confirming the content the validity of the scales in both groups. A mixed methods study may be useful to better analyze symptom and caregiver information in the future.

### Recommendations

It is worth noting that caregivers do not use the SCoRS themselves to rate the cognitive impairment but rather inform the interviewer. Interviewers will then rate the patient based on the feedback provided by the informants. It may be helpful to ask the informant to provide their view directly into the scale rather than use the interviewer to vet the informants’ feedback.

The SCoRS is an interview-based assessment that requires input from patients and caregivers. However, the evidence indicates that caregivers’ assessment may differ from self-reported evaluation of individuals with schizophrenia^32^. Therefore, clinicians completing the assessment should carefully consider the evidence when completing the SCoRS.

Although the aim of the study was to confirm the content of the SCoRS to assess cognitive impairment in patients with schizophrenia, several quotes suggest that caring for this patient population is burdensome for caregivers, due to the patient dependence and need of continuous support (e.g., repeating instructions, checking tasks, etc.). It would be useful to further explore the impact of caring for patients with schizophrenia on caregivers’ daily activities.

## Conclusion

The SCoRS is an interview-based assessment designed to evaluate cognitive impairment and the day-to-day impact of cognitive impairment in patients with schizophrenia. This non-interventional qualitative study was designed to confirm the content of the SCoRS and provide evidence to support the perceived relevance of the cognitive impairment in patients with schizophrenia that caregivers observe. Overall, the findings of this study confirm the content of the scale and support the relevance and clarity of instructions, domains, and items with primary and secondary informants caring for patients with schizophrenia.

## Supplementary information

Supplementary material

## Data Availability

Parexel was contracted by Boehringer Ingelheim to conduct the analyses, interpret the results, as well as write, review and revise the manuscript. To ensure independent interpretation of clinical study results and enable authors to fulfill their role and obligations under the ICMJE criteria, Boehringer Ingelheim grants all external authors access to clinical study data pertinent to the development of the publication. In adherence with the Boehringer Ingelheim Policy on Transparency and Publication of Clinical Study Data, scientific and medical researchers can request access to clinical study data when it becomes available on Vivli - Center for Global Clinical Research Data, and earliest after publication of the primary manuscript in a peer-reviewed journal, regulatory activities are complete, and other criteria are met. Please visit Medical & Clinical Trials | Clinical Research | MyStudyWindow for further information.
