# Supplementary material for: Concept confirmation of the Schizophrenia Cognition Rating Scale (SCoRS) among unpaid and professional caregivers
Source: Schizophrenia (Heidelb). 2025 Oct 17;11(1):126. doi: 10.1038/s41537-025-00674-2 (PMC12534470; doi:10.1038/s41537-025-00674-2)
Supplement: Supplementary file 1 — Supplementary material [file 41537_2025_674_MOESM1_ESM.docx]

**Supplementary Material**

**S1. Inclusion criteria for primary and secondary informants.**

| Inclusion criteria | Primary informants | Secondary informants |
| --- | --- | --- |
| Aged ≥18 years | √ | √ |
| Know the patient well and had been capable of interacting with the patient on a regular basis | √ | √ |
| Interacted with the patient a minimum of 1 hour per week and, preferably, at least 2 times a week. At least one interaction per week should have been in person. | √ | √ |
| Able to read, write, and speak English to complete the consent process and participate in an interview. | √ | √ |
| Willing to provide written informed consent to participate in the study | √ | √ |
| Willing to participate in a one-time interview for approximately 90 minutes via computer video conference (e.g., Microsoft Teams, WebEx) AND has access to the necessary computer equipment and internet access | √ | √ |
| Exhibit reliability and physiologic capability (e.g., sufficient hearing, vision etc.), to comply with all protocol procedures, in the investigator’s opinion | √ | √ |
| Have an educational level of minimum 8^th^ grade | √ | N/A |
| Be a health or social care professional with formal training interacting with the patient regularly on a professional basis, like a case manager, nurse, social worker, or therapist. | N/A | √ |

√ indicates that the inclusion criteria were applicable to both primary and secondary informants. N/A: not applicable.

**S2. Summary table of patient characteristics of patients from primary and secondary informant interviews.**

| **Patient characteristics** | **Patients from primary informant interviews**  Total N = 20 | **Patients from secondary informant interviews**  Total N = 20 |
| --- | --- | --- |
| **Patient cognitive impairment severity in the past 2 weeks** |  |  |
| None | 2 (10) | 0 (0) |
| Mild | 6 (30) | 7 (25) |
| Moderate | 6/7* (30/35) | 13 (65) |
| Severe | 5/6* (25/30) | 0 (0) |
| **Patient setting** |  |  |
| Inpatient | 0 (0) | 6^1^ (30) |
| Outpatient | 5 (25) | 11^2^ (55) |
| Home care | 5 (25) | 5^3^ (25) |
| Community-based (lives at same home) | 7 (35) | 0 (0) |
| Community setting (lives independently) | 3 (15) | 1^4^ (5) |
| Treatment setting | N/A | 2^5^ (10) |
| **Hospitalization for schizophrenia in the past year** |  |  |
| Yes | 6 (30) | 8 (40) |
| No | 14 (70) | 12 (60) |
| **Length of time in hospital (weeks) in the past year; Mean (range)** | 1.66 (0.28 – 3)  N = 6 | 18.27 (1.42 – 39.28)  N = 8 |
| 0 - 5 | 6 (100) | 2 (25) |
| 6 – 10 | 0 (0) | 1 (12.5) |
| 11 - 15 | 0 (0) | 1 (12.5) |
| 16 - 20 | 0 (0) | 0 (0) |
| 21 + | 0 (0) | 4 (50) |
| **Current antipsychotic medication** |  |  |
| Yes | 16 (80) | 20 (100) |
| No | 4 (20) | 0 (0) |

*One patient’s impairment was described as moderate or severe

^1^one received care at home and inpatient setting

^2^one received care in outpatient and home setting/ one receives care in home, community and outpatient setting

^3^one received care in outpatient and home setting/ one receives care in home, community, and outpatient setting/ one receives care in home and inpatient setting/ one receives care in home and treatment setting

^4^one received care in home, community, and outpatient setting

^5^one received care in treatment and home setting

N/A = Not applicable

**S3. Cognitive debrief illustrative quotes.**

*Quotes in full. Those in italics are indicative of difficulty.*

| **Aspect of debriefing** | **Primary informants** | | **Secondary informants** | |
| --- | --- | --- | --- | --- |
|  | **Agreement**  **n/N^1^ (%)** | **Illustrative quotations** | **Agreement**  **n/N^1^ (%)** | **Illustrative quotations** |
| Item 1 “Remembering names” | | |  |  |
| Correct interpretation | 17/20 (85.0%) | P6: “So does my son have difficulty remembering names of people that he knows well, friends, nurses, doctors?”    P20: “Can the person remember people, roommates, family, friends. Do they have a memory problem or short-term memory?”    *P9: “If my son has trouble communicating with people.”* | 19/20 (95.0%) | S11: “It asks me if the patient has difficulty remembering names or faces of people they’ve met, and in our clinic or the environment they’re in with us.”    S13: “The patient’s ability to remember specific people in their life.”    *S15: “If he can remember who he interacts with. In his environment around him.”* |
| Ease of response selection | 15/20 (75.0%) | P8: “That’s fairly easy.”    P13: “Well, at least that one is easy. It’s straightforward.”    *P1: “Yeah, that’s where the difficulty is because* ***he remembers all names of people that he knows****.* ***But I don’t know if he knows or remembers names of people he has just met****, so I think it’s going to be none, no difficulties.”*    *P16: “For me, it’s a little bit difficult because she does not come in contact with many people,* ***so I’d want to have like an option to say not applicable****.”*    *P20: “It’s moderate – I guess in the middle.* ***I can’t answer for her.  I don’t know what she thinks****.  I just know she knows my name, and my family, and people that she knows name.  I guess it’s difficult.  I would say it’s difficult for me to answer that question.”* | 19/20 (95.0%) | S1: “It seems easy to pick a response from those. (…) Well, it’s very straightforward. It’s like if the client can’t remember names or the client can remember names or some names, it’s very direct in the way the question is asked.”    S15: “Very easy.” |
| Examples considered helpful | 15/18 (83.3%) | P3: “The provided examples are helpful because it gives a range.”    P12: “It’s very helpful because, like you said, roommate, family and nurses. So definitely, that means those people who are close to her, or those people she meets frequently.” | 19/19 (100.0%) | S4: “I think nurse is really helpful because schizophrenia is mostly in the hospital and if you only ask about their past experiences like something – before they hospitalized that is a little hard, but when you mention nurse or some of the staff, which also caught my attention as well. I can recall some memories of this.”    S20: “That’s helpful, because it gives me a range of what you’re wanting.” |
| Item 2 “Remembering how to get places” | | |  |  |
| Correct interpretation | 18/20 (90.0%) | P3: “It asks about the level of difficulty a person has with getting places.”    P12: “That question means or is asking – Does she forget to get to rooms or places she’s used to, or maybe she frequently visits?”    P20: “Locating, getting to places, I guess locations, getting to a room, bathroom, friend’s house.” | 20/20 (100.0%) | S8: “It’s asking me if the person I’m supporting has trouble getting to the restroom or a friend’s house, and this person doesn’t have any trouble with that.”    S11: “If this patient is having difficulty remembering how to get places in their life. Again, so that’s what it’s asking me to grade my experience with that person in the last 2 weeks to that question.”    S16: “The patient’s difficulty getting to places or remembering how to get to places such as their bathroom, bedroom or friend’s house.” |
| Ease of response selection | 16/20 (80.0%) | P2: “I would think pretty easy.”    P19: “It’s pretty easy. I believe I can do it.”    *P8: “It’s a little more difficult because this is something he would be doing when I wasn’t with him. So he probably wouldn’t tell me, but you know, he may go to a bar to see live music or something like that, and he’ll get himself there, walk to the place, and I guess I don’t really know if that’s hard for him, if he uses a GPS on his phone. I don’t really have a good sense of that type of thing, it’s difficult for him. I do know that for him to walk down to his clinic is not a problem. He can do that, and that’s the other place he generally goes, but other than those two examples, he doesn’t really go anywhere.”* | 20/20 (100.0%) | S3: “It’s not difficult so it’s easy.”    S19: “I think that’s easy with the examples given, for them to choose which one to pick.” |
| Examples considered helpful | 18/19 (94.7%) | P9: “Oh, very helpful.”    P16: “I like that you gave the suggestions, because if they had just asked me is she able to get to places.” | 19/20 (95.0%) | S8: “They are good. Well, restroom, I don’t know how helpful that is. But, yeah, okay, roommates. Do you remember how to get to the hospital, that’s probably a good place. Just a thought.”    S20: “No, I was just saying, it’s helpful because it’s giving me direct examples.” |
| Item 3 “Following a TV show” | | |  |  |
| Correct interpretation | 20/20 (100.0%) | P8: “Yeah, this is asking if he can focus on a TV show, or a newscast, and I would say he does have difficulty with that.”    P14: “If the patient has some difficulties in concentrating on TV shows.”    P19: “It’s asking me how good he is with focusing on a TV show or the news.” | 18/20 (90.0%) | S6: “If the patient is able to follow the TV that they watch.”  S14: “Asking, is the person able to sit down and see a show, understand what he’s watching, and follow it. Like, you watch a show, and last week, the person, the star of the show was in a car accident. And then next week, this is this. Could he jump in and watch a show and be able to understand what’s going on? Like, know the characters, know what this TV show about, that it’s not like, “I just see this show for the first time today.”    S15: “A subject’s ability to stay focused on a TV show or program.” |
| Ease of response selection | 16/19 (84.2%) | P14: “Easy”    P16: “It’s easy to pick a response. You gave time limits on it, yeah.” | 19/20 (95.0%) | S2: “No, it’s pretty easy. It’s pretty simple, that’s why I gave it an example of the soap opera compared to watching the news. So, it’s pretty much easy to respond to it.”    S20: “Oh, it was easy to pick a response.” |
| Examples considered helpful | 20/20 (100.0%) | P3: “The provided examples are helpful.”    P12: “Those examples, they are helpful in such a way that – like a TV show or a news, it means that she needs to concentrate fully so as to understand. So, they have me to rate how difficult it has been for her, the period that has passed.” | 18/19 (94.7%) | S10: “They’re good.”    S19: “I think that’s clear for them. I think it’s helpful” |
| Item 4 “Remembering where they put things” | | |  |  |
| Correct interpretation | 20/20 (100.0%) | P10: “Like his short-term memory, if he knows where he’s putting his car keys, stuff like that.”    P4: “Does the patient have difficulty remembering where he puts things – has put things.”    P5: “How difficult it is for her to remember where has put things, like clothing or other articles, like cigarettes or whatever.” | 20/20 (100.0%) | S6: “The ability to recall their actions, what they were doing.”    S9: “Asking if the patient has any difficulty remembering where they placed everyday objects.”    S11: “It asks about the patient’s memory, specifically where they put things, if they have an issue with recalling that.” |
| Ease of response selection | 17/20 (85.0%) | P2: “Easy, those are good responses.”    P6: “It’s easier with these choices.”    P10: “It’s easy.”    *P12: “That is somewhat difficult.”* | 16/20 (85.0%) | S2: “Very easy.”    *S4: “This one is a little hard for me to pick.”*    *S20: “Okay, so the way that the question is set up and the way that those answers are set up, they’re very – the examples of the question is very specific, like cigarettes, whatever, blah, blah, blah, but it doesn’t indicate that there’s anything important or not important. Those responses and the questions aren’t really matching because you’re saying things of importance, but the examples you give don’t indicate whether something is important or not important.”* |
| Examples considered helpful | 20/20 (100.0%) | P13: “They’re helpful.” | 19/19 (100.0%) | S10: “They are helpful.” |
| Item 5 “Remembering their chores and responsibilities” | | |  |  |
| Correct interpretation | 18/20 (90.0%) | P4: “Does this person remember chores, appointments, for example, or things that he has to do?”    P5: “The level of difficulty in remembering chores that she needs to do or appointments that have been made.” | 20/20 (100.0%) | S2: “It’s asking me if the person have problem with simple choices that they have to do, keeping up their appointments. So, it’s a pretty clear question to ask.”    S13: “This question asks about patient’s memory, but also patient’s ability to take on responsibilities.” |
| Ease of response selection | 18/19 (94.7.%) | P12: “That one is easier because if you say, for the moderate option, there is an example that states the responsibilities that she will get.”    P8: “It’s fairly easy.”    P12: “That one is easier because if you say, for the moderate option, there is an example that states the responsibilities that she will get.” | 19/20 (95.0%) | S7: “Not difficult.”    S8: “Easy.    S12: “Again, as long as the descriptors are read, I think it’s easy. It still might be helpful to have a little bit of percentages under there or something like that. But overall, it’s fine.” |
| Examples considered helpful | 20/20 (100.0%) | P9: “Oh, very good. They’re good.”    P15: “The examples were very helpful.” | 17/19 (89.5%) | S3: “The provided examples are helpful.”    S10: “They’re good.” |
| Item 6 “Learning how to use new gadgets and equipment” | | |  |  |
| Correct interpretation | 20/20 (100.0%) | P1: “Does the patient has difficulty using computerized equipment.”    P5:” The level of difficulty in working with new gadgets, technology, phones, microwaves, that type of thing, computers.”    P15: “The question is about, when provided with maybe a new gadget, will she be able to operate, that she’ll know how it is used.” | 19/20 (95.0%) | S6: “A patient’s ability to learn how to use devices that would impact their adult daily living needs.”    S11: “It’s asking if the patient has trouble using equipment, devices. Yeah. So, that’s what it’s asking.” |
| Ease of response selection | 17/20 (85.0%) | P7: “Easy.”    P13: “It’s easy.” | 19/20 (95.0%) | S12: “I think those are good responses. Those are easy.”    S16: “I think it’s easy to pick a response on that.“ |
| Examples considered helpful | 19/20 (95.0%) | P10: “They’re helpful.”    P12: “It’s helpful because those are the things that – she’ll always be having them.” | 19/20 (100.0%) | S1: “Very helpful.”    S4: “The examples are helpful when asking about, you know, telephone or something. I think it’s very helpful.” |
| Item 7 “remembering information/instructions recently given to them” | | |  |  |
| Correct interpretation | 18/20 (90.0%) | P5: “Her difficulty in remembering information that she’s just learned, like telephone numbers or names of people, directions.” | 18/20 (90.0%) | S3: “If the patient has a difficult time remembering things that were recently given to him or her.”    S8: “It asks whether you have difficulty remembering names, telephone numbers, information that’s just given to you. I guess that’s like short-term memory, so do you have any difficulty with those things.” |
| Ease of response selection | 18/20 (90.0%) | P10: “Easy.” | 20/20 (100.0%) | S6: “Easy.” |
| Examples considered helpful | 20/20 (100.0%) | P2: “They’re helpful.”  P19: “It’s helpful.” | 17/17 (100.0%) | S4: “The examples make sense, it’s helpful.”    S15: “Very helpful.” |
| Item 8 “Remembering what they were going to say” | | |  |  |
| Correct interpretation | 17/20 (85.0%) | P2: “It ask if my son has difficulty communicating because he forgets words or his train thought.”    P5: “Her difficulty in completing her thoughts, her sentences. Like stopping in the middle of a sentence.”    P11: “It has to do with stopping mid-sentence and interrupting their words.” | 19/20 (95.0%) | S3: “If the patient has a difficult time remembering what they have to say.”    S9: “I understand. So the question was asking how difficult it is for the patient to remember what they were going to say, remembering words and sentences.” |
| Ease of response selection | 19/20 (95.0%) | P8: “It’s fairly easy.”    P9: “It’s easy.” | 19/20 (95.0%) | S2: “Easy.”    S5: “I would say easy.”    S9: “Easy.” |
| Examples considered helpful | 19/19 (100.0%) | P6: “(…) Yeah, they are helpful.”    P13: “Very helpful.” | 19/19 (100.0%) | S6: “Very helpful.”    S16: “They’re pretty good.” |
| Item 9 “Keeping track of their money” | | |  |  |
| Correct interpretation | 19/20 (95.0%) | P2: “It ask about my son’s ability to keep track of his money.”    P4: “Does the person have difficulty handling money, counting change and that kind of thing, paying bills?” | 19/20 (95.0%) | S1:” It’s asking me in my own words about how the illness is affecting their finances.”    S3: “If the patient has a difficult time keeping up with their finances or money. Their tangible money.” |
| Ease of response selection | 17/20 (85.0%) | P7: “Easy.”    P20: “It’s easy.” | 19/20 (95.0%) | S12: “I think it’s pretty easy as long as you read those responses with the descriptors again.”    S15: “It’s easy.” |
| Examples considered helpful | 19/19 (100.0%) | P8: “They’re helpful.”    P14: “Helpful” | 15/16 (93.8%) | S7: “Very helpful.”    S19: “Helpful.” |
| Item 10 “Keeping their words from being jumbled together” | | |  |  |
| Correct interpretation | 18/20 (90.0%) | P6: “To rate it does he have a difficult time keeping his words from being all mixed up?”  P8: “It’s asking if he gets his words mixed up and confused.”  P14: “If my patient has a problem in mixing up the words he wants to say.” | 19/20 (95.0%) | S4: “Whether you can understand what he said or not based on like healthy talk, whether he jumbled or he mixed the words together or not.”    S11: “It’s asking if the patient has difficulty, again, with speaking, keeping the words clear though, communicating clearly.” |
| Ease of response selection | 20/20 (100.0%) | P1: “That’s easy.”    P11: “Easy.” | 19/20 (95.0%) | S2: “Very easy.”    S20: “That’s very easy to pick a response.” |
| Examples considered helpful | 17/18 (94.4%) | P5: “Helpful.”    P12: “It is helpful. At least you can understand the exact question or what it means.” | 17/17 (100.0%) | S5: “I would say helpful”    S14: “Helpful.” |
| Item 11 “Concentrating well enough to read a newspaper/book” | | |  |  |
| Correct interpretation | 17/20 (85.0%) | P1: “Does the patient have quite difficulty reading or retaining information?”    P5: “Her difficulty in reading and understanding what she’s reading, a newspaper, book.”    *P19: “In my own words it’s basically asking how well does he do with concentrating on things?”* | 17/20 (85.0%) | S2: “It’s asking me if the patient or person have the ability to comprehend what they read. Do they have a problem reading certain things which is going to have them to read over and over again to relate or get the information that they need from whatever they are reading?”    S7: “Is the client able to focus, understanding what they’re reading in a way?”    *S19: “If they’re able to finish reading something and remember what they read.”*    *S20: “It sounds like if they are able to kind of, I guess, flow with what they’re doing as opposed to the need to repeat or like they’re getting stuck.”* |
| Ease of response selection | 16/19 (84.2%) | *P2: “That might be a little tricky for me and it seems like there should be something in between severe in the next response down. That would make it simpler for me.”*    P7: “Easy.”    P9: “It’s not hard. It’s not hard. I’m trying to picture him when he’s reading or doing things, and I know sometimes it’s harder than others.” | 19/20 (95.0%) | S14: “I think it’s easy.”    S15: “Easy.”    S18: “Easy.” |
| Examples considered helpful | 20/20 (100.0%) | P2: “Very helpful.”    P16: “Helpful.” | 17/17 (100.0%) | S2: “Very helpful.”    S15: “They’re helpful.” |
| Item 12 “Familiar tasks” | | |  |  |
| Correct interpretation | 18/20 (90.0%) | P10: “Talking about a daily function, like hygiene and stuff like that, like everyday things.”    P13: “If the patient is able to handle household tasks or take care of themselves.”    P16: “In the last two weeks, has my daughter had any difficulty with tasks of everyday living.” | 19/20 (95.0%) | S2: “You ask about if they are able to do normal grooming for themselves. They don’t have any problem. They are able to take a shower. They are able to groom themselves, but other than that, everything else is very limited towards them.”    S8: “Daily chores, daily living. It asks about can you cook dinner, can you drive if you have a car. Or do you have difficulty with that?” |
| Ease of response selection | 17/20 (85.0%) | P6: “It was easy.”  *P8: “It’s a little difficult because, again, I think most of these things he’s doing when he’s alone, so I’m not really witnessing it.”*  P16: “It’s easy. The only thing for me is that once again, it’s one of those things like one task there’s none, and then the other one might be moderate, do you know what I’m saying, because with different tasks, they might have different needs with it, so I’m just saying that.” | 20/20 (100.0%) | S3: “Easy.”    S17: “It’s relatively easy.” |
| Examples considered helpful | 18/19 (94.7%) | P8: “They’re helpful.”    P13: “Helpful.” | 18/18 (94.4%) | S7: “Very helpful.”    *S14: “That example is a little different.”*    *INTERVIEWER: “All right.  Could you please just walk me through that?”*    *S14: “Because I’m not sure if I’m giving the right response.  It’s asking about getting dressed and stuff like that.  I’m thinking more so like daily grooming.  And like I said, I’ve never witnessed the person get dressed or anything like that, just that they always appear neat and clean in appearance.  But also, clean appearance doesn’t mean that someone’s showered.  So, that one’s a little challenging for me.”* |
| Item 13 “Staying focused” | | |  |  |
| Correct interpretation | 16/20 (80.0%) | P10: “If he’s paying attention, not being distracted.”    *P1: “If the person has hard time understanding others.”*    *P13: “If they have hallucinations.”* | 20/20 (100.0%) | S1: “In my own words, it’s asking me what’s going on cognitively in terms of thought blocking, are they really able to follow a conversation and interact with another person in a way that, like it came to my mind is meaningful, but that’s not what I mean. What I mean is like, how present they really are in the interaction with them and are they really responding to what’s going on with us. Are they responding what’s going on in their head or internally.”    S2: “Does the person have any problem staying focused, concentrating on things or anything that’s going to cause them some kind of discomfort of just to think.” |
| Ease of response selection | 17/20 (85.0%) | P6: “It was very easy.”    P15: “Not difficult.” | 20/20 (100.0%) | S3: “Easy to pick a response.”    S20: “I guess, easy to pick a response.” |
| Examples considered helpful | 20/20 (100.0%) | P7: “Fine, they’re good.”    P11: “Very good.” | 18/18 (100.0%) | S1: “The examples are very helpful.”    S14: “The examples are good.” |
| Item 14 “Learning new things” | | |  |  |
| Correct interpretation | 18/20 (90.0%) | P12: “That’s more about her ability to learn, how easy it is for her to learn new things, maybe which she has never seen before, or maybe which she has seen before, but they are modified to make them look new.” | 19/20 (95.0%) | S3: “If the patient has difficulty with managing basically new information. So, scheduling – all the things that you listed here.” |
| Ease of response selection | 16/20 (80.0%) | P5: “Okay, all right, fairly easy to rate that now.”    *P16: “I thought it was a little bit harder to pick one because of the fact that when you’re learning new things, it’s not always the same, I guess.”* | 20/20 (100.0%) | S7: “Easy. “    S16: “Very easy.” |
| Examples considered helpful | 18/18 (100.0%) | P10: “They’re helpful”    P17: “Helpful” | 18/18 (100.0%) | S9: “Very helpful”    S19: “I think it’s pretty helpful to understand.” |
| Item 15 “Speaking as fast as they would like” | | |  |  |
| Correct interpretation | 20/20 (100.0%) | P9: “If [REDACTED] has the ability to communicate, and I don’t see any problem in that area at all.”    P14: “If my patient has problems with speaking faster.”    P17: “Does he have trouble speaking as fast as he would like” | 17/20 (85.0%) | S3: “If the patient has difficulty speaking at a regularly paced conversation speed, I guess. Yeah, that’s it.”    S4: “Asking about their speed of talking.”    *S13: “Verbal fluency and speech patterns.”*    *S19: “Them being able to say what’s on their mind.”* |
| Ease of response selection | 18/20 (90.0%) | P5: “Easy.”    P12: “It’s easier because every response is binding or embedded to the condition. So, it will keep my focus on the condition only, not focusing on other things.” | 19/20 (95.0%) | S3: “Easy”    S11: “It’s easy.” |
| Examples considered helpful | 18/19 (94.7%) | P8: “They’re helpful.”    P12: “Somewhat helpful because when you say about speech, I’ll be able to know it is her language.” | 18/18 (100.0%) | S6: “Very helpful.”    S13: “Helpful.” |
| Item 16 “Doing things quickly” | | |  |  |
| Correct interpretation | 14/20 (70.0%) | *P1: “Does the patient have difficulty doing normal activities.”*    *P10: “Again, I would say it’s more being able to connect the dots.”*    *P13: “If the patient is capable of completing simple tasks.”*    P14: “If my patient has a problem of doing things quickly.” | 16/19 (84.2%) | *S1: “That’s important I guess in my own words because if they’re not able to do that, that will give me an idea of how frustrated they get. Then I look to whether they’re able to manage that frustration. But it tells me their ability to really act in the moment in a way that is useful or helpful to them.”*    S8: “Do you have difficulty doing things quickly, such as lighting your cigarette or writing.”    *S14: “Does this person have problems on executing on-demand, like you got a cigarette in your mouth, will they just stand there and hold it, or they just go ahead and light it?  Problems like that, so are they able to, I guess, execute on demand?”*    *S15: “What’s the subject’s difficulty or lack of to complete small tasks.”* |
| Ease of response selection | 14/20 (70.0%) | P6: “It was pretty easy.”    *P9: “Well, between the two, it’s a little difficult. (…) Depends in different tasks. It depends on the type of task.”*    *P13: “A bit difficult. (…) It’s rare for her to do such things like writing.  So, I’m not that much – from a young adult – I’ve not experienced that.”* | 20./20 (100.0%) | S1: “It’s easy.”    S17: “Moderately easy.” |
| Examples considered helpful | 15/20 (75.0%) | P10: “They’re helpful.”    *P2: “Those examples are kind of not very helpful because those are too way different things. Writing is far more difficult than lighting a cigarette. So, I would rate those as bad examples to throw together.”* | 19/19 (100.0%) | S7: “Very helpful.”    S11: “They were helpful.” |
| Item 17 “Handling changes in their daily routine” | | |  |  |
| Correct interpretation | 17/20 (85.0%) | P5: “Her difficulty in handling changes that happen in scheduling, like appointments and different day-to-day activities.”    P9: “If [REDACTED] follows up on all his appointments and things he needs to do.”    *P20: “Can my niece do tasks that’s going to her everyday – well, completing tasks as far as going into group therapy and being productive.”* | 19/20 (95.0%) | S3: “If a patient has a difficult time with managing change.”    S6: “This question is asking about a patient’s ability to adapt to sudden change.”    S19: “Does the person that I care for have difficulty with accepting changes to the routine?” |
| Ease of response selection | 17/20 (85.0%) | *P6: “It would be difficult for me because I’m between none and mild. Because this doesn’t really happen to him that much. He obviously doesn’t have group therapy. I mean, he can handle the change. Would he go to the appointment? I don’t know.”*  P13: “Easy.” | 20/20 (100.0%) | S7: “Easy.”    S12 “It’s not bad. Just – what does ‘considerable effort’ mean? How do we define that? It’d be good to have a little bit more clarity with that.” |
| Examples considered helpful | 17/18 (94.4%) | P6: “They are helpful.”    P18: “Helpful” | 18/18 (100.0%) | S4: “The examples are good.”    S15: “They’re helpful.” |
| Item 18 “Understanding what people mean when they are talking to them” | | |  |  |
| Correct interpretation | 19/20 (95.0%) | P4: “Does he have difficulty understanding what someone says to him?”    P8: “It’s asking if he’s able to understand what people are saying to him.”    P20: “Can my niece understand what people are saying to her, or is she confused when people are talking?” | 19/20 (95.0%) | S1: “It’s asking me how they interpret what other people are saying. Are they in the ballpark of the conversation or that they go places that can really cause problems. That’s the way I hear it.”    S3: “If the patient comprehends what was asked to them or what is being communicated to them.”    S19: “If they understand what’s being said to them.” |
| Ease of response selection | 18/20 (90.0%) | P6: “It was pretty easy.”    P12: “It would be easier to pick a response because I will now be in a position to think about how she communicates with others, so it’s easier.” | 19/20 (95.0%) | S6: “Easy”    S15: “It is easy.” |
| Examples considered helpful | 17/19 (89.5%) | P5: “Helpful.”    P7: “They’re pretty helpful. Fine.” | 17/18 (94.4%) | S7: “Very helpful.”    S13: “Helpful.” |
| Item 19 “Understanding how other people feel about things” | | |  |  |
| Correct interpretation | 19/20 (95.0%) | P2: “It ask about my son’s ability to pick up on cues to read other people’s feelings.”    P4: “Yeah. Does he have difficulty understanding people’s emotions, or what they’re feeling or thinking? Yeah.”    P16: “In the last two weeks, has my daughter had difficulty understanding how I feel about situations.” | 19/20 (95.0%) | S4: “Their interaction ability or the ability to recognize others’ feelings, identifies others’ feeling, emotions.”    S11: “It’s asking about the patient’s ability to pick up on body language.”    S12: “Does the participant have difficulty understanding people’s emotions?” |
| Ease of response selection | 16/20 (80.0%) | *P1: “It wasn’t easy. It was between mild and moderate, so it was a little bit more difficult to pick a response for that one.*    *P8: “I’m not always sure what he is thinking.  I mean, it’s hard to say if he has misunderstood the way people are feeling or I’m just – he hasn’t expressed it to me, so I’m not always sure what he’s thinking.”*    P10: “Easy.”  *P11: “I think it’s mild, and the question is not so easy to answer. (…) Because I’m not so sure about the answer.  I’m not so certain about how she interprets that at the moment.  I think she’s pretty good with it.  In general, she interprets very well.  But there may be just some mild, you know.”* | 19/20  (95.0%) | S2: “It is very easy.”    S5: “I would say easy.”    S7: “Easy.” |
| Examples considered helpful | 20/20 (100.0%) | P12: “The examples, they’re useful or helpful because they cover communication as a whole, both using words and non-verbal cues.”    P16 “They’re helpful.” | 18/18  (100.0%) | S3: “The provided examples are helpful.”    S10: “Helpful.” |
| Item 20 “Following conversations in a group” | | |  |  |
| Correct interpretation | 19/20 (95.0%) | P5: “Your ability to follow multiple conversations in a group setting.”   P11: “Do they have difficulty following participation in a group?”    P18: “Does he have problems with following a conversation within a group of people, I guess, or a socialized group.” | 20/20 (100.0%) | S3: “If a patient is able to follow talking to multiple people at once or interacting with multiple people at once.”    S6: “This question asks about a patient’s ability to engage with group activities, with conversations and to be focused while in group settings.”    S20: “Are they able to engage, but like I said, is that going to be more effective if asking like can they shift attention between people, because to – like engage in – like with group conversation, it seems like it should be, the examples, like one of them sounds like it should be “can they shift attention to speakers” or something. Like that part seems like it should be an example.” |
| Ease of response selection | 16/20 (80.0%) | *P1: “That was harder.  It was difficult.”*    P7: “That was easy”    *P16: “I think this one is a little bit hard in that, once again, we’re trying to decipher whether she is following or not. I think the severe one would be kind of easy, because things probably are pretty clueless, but sometimes you don’t know whether they’re following or not.”* | 20/20 (100.0%) | S2: “Very easy.”    S16: “Those are very easy.” |
| Examples considered helpful | 19/19 (100.0%) | P6: “Very helpful.”    P20: “Very good.” | 18/18 (100.0%) | P8: “That’s good, yeah. Yeah, good.”    S14: “Helpful.” |
